# An effective deep learning algorithm for medical image registration

**DOI:** 10.1371/journal.pdig.0001339

**Published:** 2026-04-16

**Authors:** Jinqiu Deng, Ke Chen, Mingke Li, Zhichao Zuo, Alejandro F. Frangi, Jianping Zhang

**Affiliations:** 1 School of Mathematics and Computational Science, Xiangtan University, Xiangtan, China; 2 Department of Mathematics and Statistics, University of Strathclyde, Glasgow, United Kingdom; 3 Radiology Department of Xiangtan Central Hospital, Xiangtan, China; 4 Schools of Health Sciences and Computer Science, University of Manchester, Manchester, United Kingdom; National Taichung University of Science and Technology, TAIWAN

## Abstract

Image registration is crucial for many medical imaging applications, including longitudinal monitoring and multimodal information fusion. A key challenge is to achieve accurate alignment while strictly preserving topology and invertibility. To address the limitations of traditional penalty-based regularization, which may still permit local folding, this study proposes DTC-Reg, a dynamically learned registration framework that more explicitly enforces diffeomorphic deformation. The framework integrates a homotopy-based control–increment formulation with explicit multiscale geometric constraints. Two parameter-sharing U-Nets first extract multiscale feature pyramids from the input images, after which a symmetric registration module with a sequential temporal cascade network progressively refines the forward and inverse multiscale deformation fields. To further enhance diffeomorphic consistency, this study introduces a Multiscale Folding-aware Deformation Correction (MFDC) module that explicitly detects and geometrically rectifies folding points in the predicted deformation fields. Beyond its integration within DTC-Reg, MFDC can also be readily incorporated into several state-of-the-art registration networks, significantly reducing folding and improving deformation regularity. Extensive experiments on three 3D brain MRI registration tasks demonstrate that the proposed method consistently achieves superior performance over existing approaches in both quantitative and qualitative evaluations.

## Author summary

Automated medical image registration is crucial for diagnosis but often suffers from unrealistic topological distortions. This study introduces DTC-Reg, a deep learning framework that ensures anatomically plausible alignment by mimicking fluid mechanics. Uniquely, it incorporates a geometric correction mechanism, MFDC, that automatically identifies and repairs topological errors. Experiments on brain MRI datasets demonstrate that DTC-Reg achieves state-of-the-art accuracy while completely eliminating tissue folding. This work provides a mathematically rigorous and reliable solution for automated clinical image analysis.

## Introduction

Deformable image registration is a crucial technique in medical image analysis that aligns anatomical structures in images [1]. This technique is essential for various clinical applications, including lesion identification [2], dose accumulation [3], motion tracking [4], and image reconstruction [5]. Traditional registration methods typically formulate image registration as a variational problem and solve it iteratively using optimization algorithms [6], such as Demons [7], B-spline [8], LDDMM [9], Diffeomorphic Demons [10], SyN [11], diffeomorphic image registration with control increment constraint [12], and their variants [13–20]. Although these approaches preserve diffeomorphism and achieve high registration accuracy, they are computationally expensive because they require time-dependent optimization as well as extensive hyperparameter tuning.

Following the success of AlexNet [21] in the ImageNet challenge, deep learning has been increasingly applied to image processing tasks and has achieved remarkable performance across a wide range of applications. In recent years, numerous deep learning frameworks have been proposed for medical image registration [1,22,23]. Initially, most neural networks for image registration required supervision from ground-truth deformation fields. More recently, unsupervised CNN-based methods have become a major focus in deep-learning-based image registration research [24–33]. Unlike traditional methods, unsupervised deep learning registration methods have remarkably improved computational speed while maintaining accuracy [34].

Current learning-based registration methods, such as VoxelMorph [24], use two concatenated images as input and apply the U-Net architecture to directly extract features and then generate deformation or velocity fields. However, these straightforward methods may lack accuracy in complex scenarios. For complex or large-scale deformations, the VTN framework [26], which uses a cascade of multiple networks, proves to be an effective approach. Typically, these cascades consist of serially connected U-Nets, each of which progressively learns the deformation field and transforms the moving image to align with the fixed image. However, this method involves high computational costs and tends to overfit. It also accumulates errors during multiple interpolations. SYM-net [28] and similar approaches have explored symmetric registration. Moreover, most existing deep learning techniques including VoxelMorph [24] and VTN [26] are limited to unidirectional registration, neglecting the invertibility of the deformation field, i.e., its diffeomorphic property. The scaling and squaring method [35] is widely adopted for diffeomorphic registration. However, this technique is limited by the assumption of a constant velocity field, which may constrain its capability to capture fine-scale deformations. Additionally, the coupling nature of scaling-and-squaring’s iterative solution can lead to interpolation errors.

Taken together, these representative methods still leave several important challenges unresolved. First, many learning-based predictors estimate deformation fields in a feed-forward manner and rely primarily on smoothness regularization, which does not explicitly guarantee topology preservation and may still permit residual folding in challenging regions. Second, diffeomorphic variants based on stationary velocity fields and scaling-and-squaring typically enforce invertibility under a constant-velocity assumption; this may limit flexibility for fine-scale or spatially heterogeneous deformations and may also accumulate interpolation errors through repeated compositions. Third, symmetric or cycle-consistency constraints improve bidirectional consistency, but they remain indirect and do not explicitly repair local topological violations once folding occurs. Finally, many existing approaches still face an accuracy–topology trade-off: stronger regularization may improve diffeomorphic behavior, but often at the cost of over-smoothing anatomically meaningful details and reducing alignment accuracy.

To address these unresolved issues, this study proposes DTC-Reg (Diffeomorphic Temporal Cascade Registration), a novel learning framework that unifies variational modeling, multiscale cascades, and symmetric registration through temporal recurrent units (e.g., ConvLSTM [36]). The proposed method consists of three key innovations:

- **Variational loss formulation:** This study adopts a loss function inspired by recent variational models, leveraging physically meaningful regularization to guide diffeomorphic matching through the entire training process. The adopted diffeomorphic control approach introduced by Zhang and Li [12] reformulates the implicitly controlled Large Deformation Diffeomorphic Metric Mapping (LDDMM) model. Their dynamic system formulation provides a direct connection to explicit diffeomorphic control, enabling more flexible and efficient learning-based registration.
- **Multiscale temporal cascade architecture:** Two parameter-sharing U-Nets extract a multiscale feature pyramid from each image pair. These features feed into a Symmetric Refinement (SR-Module) with a temporal cascade structure that iteratively refines both forward and reverse deformation fields. By enforcing symmetry and pre-warping features at each scale, this architecture implements a multiscale registration strategy that improves both convergence and alignment accuracy.
- **MFDC (Multiscale Folding-aware Deformation Correction):** To ensure topological consistency and preserve diffeomorphism, this study introduces MFDC, which combines multiscale folding-aware fusion with a polygon-based geometric correction mechanism. MFDC imposes explicit geometric constraints on the deformation field to prevent folding and singularities, even at fine resolution levels.

Together, these components enable DTC-Reg to deliver accurate, symmetric, and strictly diffeomorphic registration in a computationally efficient, end-to-end trainable framework.

## Related work

This section provides a brief overview of model-based and data-driven approaches to image registration.

### Diffeomorphic Image Registration

The most challenging type of medical image registration is deformable image registration (DIR), especially diffeomorphic DIR. When optimizing an energy function, diffeomorphic DIR establishes the spatial transformation relationship between two images. Let *X* and *Y* represent the moving and fixed images, diffeomorphic deformation field $\hat{\phi }$ of image registration is determined by minimizing an energy function as


$$\hat{\phi }=\underset{\phi \in \text{Diff}(\Omega )}{\arg min}{ℒ}_{sim}(X,Y\circ \phi )+\lambda {ℒ}_{smooth}(\phi ),$$ (1)

where Ω denotes the spatial domain of the images and Diff(Ω) denotes the set of diffeomorphisms on Ω (i.e., smooth and invertible transformations with smooth inverses). The operator “∘” denotes composition (warping), so Y∘ϕ represents the warped image. The term ${ℒ}_{sim}(X,Y\circ \phi )$ measures image similarity between *X* and Y∘ϕ, while ${ℒ}_{smooth}(\phi )$ is a regularization term that encourages spatial smoothness of ϕ. The scalar λ>0 controls the trade-off between similarity and smoothness. Since ϕ is diffeomorphic, it admits an inverse mapping $\phi^{-1}$ satisfying $\phi \circ \phi^{-1}=\phi^{-1}\circ \phi =𝕀$, where 𝕀 denotes the identity mapping on Ω.

### Image registration via deep learning

Deep learning methods use data-driven networks to align images. VoxelMorph, created by Balakrishnan et al. [24], uses the U-Net architecture to achieve accurate image alignment. However, the quality of the deformation field was not optimal. To address this issue, Dalca et al. [25] improved VoxelMorph by incorporating scaling and squaring techniques [35]. They decomposed the deformation field into integrals of multiple velocity fields and derived the final approximate diffeomorphic deformation field through integration. This approach has been extensively adopted in later research concerning diffeomorphic registration using deep learning techniques.

The scaling and squaring technique in medical image registration has led to multiple frameworks. VTN [26] decomposes large displacements into smaller ones and then uses cascaded U-Net networks to refine the registration process from coarse to fine levels. CycleMorph [27] employs cycle loss as an implicit regularization to aim for diffeomorphic registration. SYM-net [28] uses symmetric deformation fields. Kang et al. [30] propose a dual-stream pyramid network that utilizes two U-Nets with shared parameters to extract features from input data at various scales. These features are then fused using a PR++ module for multi-resolution registration. Wei et al. [31] incorporate an adaptive smoothing layer and an anti-folding constraint into the U-Net based registration network. Chen et al. [37] address the challenge of limited connectivity in long-range spatial interactions within a CNN network by using TransMorph, which combines Vision Transformer with CNN.

Inspired by quasi-conformal (QC) Teichmüller theories, Chen et al. [38] proposed a deep learning framework to learn the Beltrami coefficient for maintaining diffeomorphic registration. Chen et al. [39] use randomized Beltrami coefficients to acquire map labels. Using QC theories, Zhang et al. [40] developed the topology preservation segmentation network to achieve object segmentation while aiming to preserve the topology of the image.

Despite these advances, most existing learning-based registration networks still rely primarily on penalty-based regularization to discourage folding. Such strategies may reduce the occurrence of folding, but they do not explicitly eliminate local topological violations, especially under large or spatially heterogeneous deformations.

### Scaling and squaring approach

Dalca et al. [25] built upon the mathematical framework of DARTEL [41] to develop a deep learning approach aiming for diffeomorphic registration. This is one attempt to implement LDDMM [9] in learning. They assume a stationary velocity field and model the evolution of the deformation ϕ(x,t) using the following differential equation:


$$\frac{d\phi \left(x,t\right)}{dt}=v(\phi \left(x,t\right)),\;t\in [0,1],$$ (2)

Starting from the identity at *t* = 0, the final deformation $\phi_{1}$ is computed by integrating a stationary velocity field v using the scaling and squaring method [35]. In this framework, the deep learning network predicts a stationary velocity field v. The velocity field is first scaled by 1/2^N^ to generate the initial deformation field $\phi_{1/2^{N}}=x+v(x)/2^{N}$, where *N* is the number of scaling steps. The deformation is then recursively composed as $\phi_{1/2^{t-1}}=\phi_{1/2^{t}}\circ \phi_{1/2^{t}}$, until $\phi_{1}$ is reached.

However, this approach generally enforces diffeomorphism indirectly through smoothness regularization of the velocity field v, without incorporating explicit mechanisms to guarantee diffeomorphic mappings during the registration process. Moreover, the constant velocity assumption in DARTEL can lead to unnecessarily complex and energy-intensive deformation paths [41]. Some diffeomorphic mappings, which would be easily attainable with time-varying velocity fields, become unreachable under this fixed-velocity formulation. Many deep learning-based registration methods [25,27,28,31,37] that adopt the DARTEL framework and the scaling-and-squaring strategy also share this limitation. Consequently, these methods may still exhibit residual folding or accuracy degradation when stronger smoothness is imposed to maintain diffeomorphism, highlighting the need for an explicit, geometry-driven correction mechanism.

### Reformulation as a dynamical system

To address the limitations of stationary velocity field models such as DARTEL and the scaling and squaring approach, Zhang and Li [12] proposed a time-dependent formulation based on homotopy continuation, which allows more flexible and expressive deformation modeling. A key insight of their work is that the diffeomorphic condition can be explicitly embedded in the formulation itself, instead of requiring smoothness constraints on the velocity field. This is achieved through a control increment u(ϕ(x,t)), where the velocity field v is defined based on equation (2) as:


$$v(\phi \left(x,t\right)):=\frac{u(\phi \left(x,t\right))}{h(\phi \left(x,t\right),t)},$$ (3)

where the homotopy function h(ϕ(x,t),t)>0 encodes a time-dependent embedding from 0 to 1. A key result in their formulation shows that the control system must satisfy the following condition to guarantee the preservation of diffeomorphism:


$$\begin{array}{c} \left\{ \begin{array}{cc} \text{div}(u(\phi (x,t)))+\frac{\partial h(\phi (x,t),t)}{\partial t}=0, & x\in \Omega_{in},\\ u(\phi (x,t))=0, & x\in \partial \Omega . \end{array}\right. \end{array}$$ (4)

where div(·) denotes the spatial divergence operator, $\Omega_{in}$ denotes the interior of the spatial domain Ω, and ∂Ω denotes the boundary of Ω. This constraint leads to a deformation ϕ(x,t) whose Jacobian determinant remains strictly positive over time, expressed as:


$$\det\nabla \phi (x,t)=\frac{h(x,0)}{h(\phi (x,t),t)}>0,t\in (0,1].$$ (5)

As designed, this approach ensures smooth, invertible, and topology-preserving mappings at all time steps. The proposed learning-based method will be built upon this formulation to guarantee diffeomorphic transformations throughout the registration process.

## Methodology

**Fig 1:**
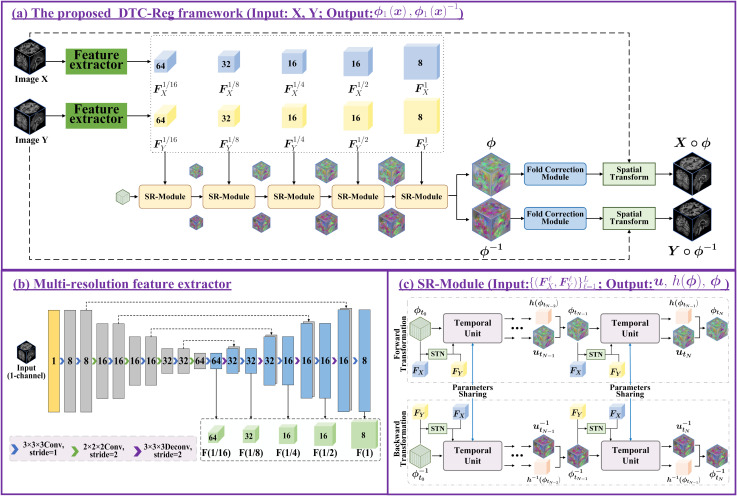
Architecture of the DTC-Reg framework for symmetric diffeomorphic registration. **(a)** DTC-Reg framework: A symmetric configuration featuring two competing pathways to ensure one-to-one deformation between images *X* and *Y*. **(b)** Dual multiscale feature extractor: Weight-sharing U-Nets generate multiscale feature pyramids $\{F_{X}^{\ell }{\}}_{\ell =1}^{L}$ and $\{F_{Y}^{\ell }{\}}_{\ell =1}^{L}$ from input pairs. **(c)** SR-Module: A cascade of *N* temporal blocks that iteratively estimates incremental fields, which are integrated to produce the final forward and backward deformation fields. For reproducibility, all tensors follow the shape *B* × *C* × *H* × *W* × *D*, where (*H*,*W*,*D*) denote the spatial size at the current scale. At each scale, the predicted control-incremental field has 3 channels (i.e., *B* × *3* × *H* × *W* × *D*), and is accumulated over *N* steps to obtain the final forward/backward deformation fields at that scale’s resolution.

This study presents DTC-Reg, a symmetric diffeomorphic registration framework illustrated in Fig 1, which is specifically designed to fulfill the modeling requirements defined in (3)–(5). Given a pair of input images *X* and *Y*, the model first extracts multiscale feature pairs at five resolution levels using two weight-sharing U-Net encoders. These features are then passed through a cascade of SR-Modules (one per scale) to progressively estimate the time-dependent control increments $\{u_{t_{n}}^{\ell }{\}}_{\ell =1,n=1}^{L,N}$, the homotopy composition $\{h(\phi_{t_{n}}^{\ell }){\}}_{\ell =1,n=1}^{L,N}$, and the corresponding deformation fields $\{\phi_{t_{n}}^{\ell }{\}}_{\ell =1,n=1}^{L,N}$, along with their symmetric inverses. These forward and inverse quantities are directly involved in both network propagation and loss computation. The final deformation field $\phi_{1}\left(x\right)$ and its inverse are obtained through the following integration:


$$\{\begin{array}{c} \phi \left(x,t\right)=\phi \left(x,t_{old}\right)+\Delta t\;\frac{u\;\left(\phi (x,t_{old}),t_{old}\right)}{h\;\left(\phi (x,t_{old}),t_{old}\right)},\\ \phi \left(x,0\right)=x. \end{array}$$ (6)

where *t*_old_ denotes the previous time step and Δt is the temporal step size, ensuring diffeomorphic evolution across the temporal domain and preserving topological consistency throughout the registration process.

The DTC-Reg architecture consists of five principal components: 1) Dual Multiscale Feature Extractor: a U-Net module for multiscale feature extraction designed to obtain dual feature pyramids; 2) Learning Deformation Increments: a learnable control increment module obtained from preregistered features using homotopy continuation; 3) SR-Module for Symmetric Multiscale Registration: an SR-Module designed for invertible symmetric path registration, which integrates with cascaded ConvLSTM blocks to learn symmetric deformation fields; 4) the MFDC Module (Multiscale Folding-aware Deformation Correction) to guarantee topological validity; and 5) Loss Function: different diffeomorphic losses and a similarity loss which are incorporated into the proposed network.

### Dual multiscale feature extractor

DTC-Reg employs two dual U-Nets with shared parameters to extract feature pyramids of moving and fixed images, as shown in Fig 1(b). The encoder comprises two 3D convolutional layers (3x3x3) with a stride of 1, followed by four additional 3D convolutional layers (2x2x2) with a stride of 2, allowing hierarchical downsampling. Each convolutional operation is followed by a ReLU activation function. In the decoder, there are four 3D transposed convolutional layers (3x3x3) with a stride of 1, employed for upsampling until the original image resolution is restored. The encoder and decoder are connected through skip connections, which are illustrated by dashed lines in Fig 1(b). The dual U-Nets share weights and extract five-level multiscale feature pyramids for both images, with channel dimensions {64, 32, 16, 16, 8} at successive scales. The feature extractor can be replaced by more advanced architectures such as Vision Transformers (ViTs) [42]. However, experiments show that U-Net is sufficient, and its lightweight design avoids unnecessary computational overhead in feature extraction.

The proposed approach differs from learning-based methods such as VoxelMorph [24] by decoupling feature extraction from deformation estimation via a variational registration mechanism. This results in more accurate and hierarchically structured deformation fields across five scales. Furthermore, the proposed method offers greater interpretability and facilitates integration with mathematical models.

### Learning deformation increments

After the pyramids of the features are extracted, the proposed DTC-Reg proceeds to learn the increment fields $\{u_{t_{n}}^{\ell }{\}}_{\ell =1,n=1}^{L,N}$ of deformation in multiple stages, and then computes the deformation fields $\{\phi_{t_{n}}^{\ell }{\}}_{\ell =1,n=1}^{L,N}$ using formula (6), as shown in Algorithm 1.

First, the dual multiscale feature extractor generates feature pyramids $\{(F_{X}^{\ell },F_{Y}^{\ell }){\}}_{\ell =1}^{L}$ from each input pair (*X*,*Y*). A multiscale registration procedure is then performed in a coarse-to-fine manner, starting from the coarsest scale and progressing to the finest scale. At each scale ℓ, DTC-Reg sequentially estimates *N* control increments $\{u_{t_{n}}^{\ell }{\}}_{n=1}^{N}$ through the proposed SR-Module and updates the intermediate deformation field $\phi_{t_{n}}^{\ell }$ following Algorithm 1 (with *t*_*n*_ and Δt defined therein) until the final deformation $\phi_{1}^{\ell }:=\phi_{t_{N}}^{\ell }$ is obtained. The resulting $\phi_{1}^{\ell }$ is then upsampled by a factor of 2 to initialize the nex*t* finer scale, i.e., $\phi_{t_{0}}^{\ell +1}$. Repeating this process across scales yields the final deformation $\phi_{1}:=\phi_{t_{N}}^{L}$.

To preserve topology throughout the cascade, the temporary deformation field ϕ(x,t) in (6) is subject to a homotopy composition denoted by h(ϕ(x,t),t) [12]. This study approximates h(ϕ(x,t),t) as


$$h(\phi (x,t),t)\approx \frac{1}{2\pi \sigma^{2}}\int_{\Omega }h(y)\exp\;\left(-\frac{\|y-\phi (x,t){\|}^{2}}{2\sigma^{2}}\right)\;dy,$$ (7)

where $h(y):=h_{0}(y)=1$ at *t* = 0, y is the integration variable, σ is the bandwidth of the Gaussian kernel, and Eq. (7) approximates h(ϕ(x,t),t) via Gaussian-weighted smoothing of h(y) centered at ϕ(x,t).

**Algorithm 1 DTC-Reg.**

**S-1** Input the moving and fixed images *X* and *Y*, initial deformation field $\phi_{0}\;(x)\;=\;x$;

**S-2** Extract dual pyramid features ${\left\{\left(F^{\ell }X,F_{{\;}_{Y}}^{\ell }\right)\right\}}_{\ell =1}^{L}$ with *L* scales from inputs (*X*,*Y*) by the dual U-Net modules of the proposed DTC-Reg, respectively;

**S-3** Use *N* cascaded ConvLSTM at each feature scale ℓ to gradually learn the deformation field from *t* = 0 with time step-length $\frac{1}{N}\;\left(n\;\leq \;N,\;\ell \;\leq \;L\right)$.

**S-3.1** Pre-align features $\left[F_{X}\circ \;(\phi_{t_{n-1}}^{\ell -1}\;\;),F_{Y}\right]$ and $\left[F_{X},F_{Y}\circ \;({\left(\phi_{\;t_{n-1}}^{\ell -1}\;\right)}^{-1})\right]$;

**S-3.2** Use cascade ConvLSTM to learn the control incremental fields $u_{t_{n}}^{\ell }$ and ${\left(u_{t_{n}}^{\ell }\right)}^{-1}$ at time $t_{n}\;=\;\frac{n}{N}$;

**S-3.3** Iteratively update the current deformation field by.

$\phi_{t_{n}}^{\ell }\;\;=\phi_{t_{n-1}}^{\ell }\;+\;\Delta t\;\cdot \;\frac{u_{t_{n}}^{\ell }}{\;h(\phi_{t_{n-1}}^{\ell })}$,

${\left(\phi_{t_{n}}^{\ell }\right)}^{-1}\;\;={\left(\phi_{t_{n-1}}^{\ell }\right)}^{-1}\;+\;\Delta t\;\cdot \;\frac{{\left(u_{t_{n}}^{\ell }\right)}^{-1}}{h({\left(\phi_{t_{n-1}}^{\ell }\right)}^{-1})}$;

**S-4** Output final solutions $\phi_{1}\;(x)$ and $(\phi_{1}\;{\left(x)\right)}^{-1}$.

Unlike the scaling and squaring approach, the proposed registration technique integrates control increments directly into the deformation field, thereby minimizing the error amplification that results from repeated interpolations. Additionally, the use of a variable velocity field enhances adaptability, enabling simple and direct corrections to the deformation field.

### SR-Module for symmetric multiscale registration

To handle cascaded registration tasks, previous work [26] proposed cascaded U-Net architectures; however, such designs are often computationally inefficient and prone to overfitting. To overcome these issues, this study introduces a SR-Module for progressive, time-aware registration. The SR-Module, illustrated in Fig 1(c), is designed to achieve symmetric deformations for both moving and fixed images through a multiscale temporal cascade framework. Its key idea lies in leveraging sequential modeling to progressively refine deformation fields across multiple spatial scales. Specifically, it learns sequential increment fields $u_{t_{n}}^{\ell }$ over five levels (ℓ=1,…,5) from coarse to fine resolution.

The SR-Module is model-agnostic and can incorporate any temporal modeling network to perform cascaded registration. It is not limited to conventional architectures like LSTM or gated recurrent unit (GRU); the temporal framework itself can be redefined to accommodate more advanced or domain-specific sequential models, making it adaptable to future developments in temporal learning. In this study, ConvLSTM [36] is used as a representative example. At the initial time step, ConvLSTM receives the extracted features $\left(F_{X},F_{Y}\right)$ along with an identity deformation field. In subsequent cascade stages, it takes the previously aligned features $\left[F_{X}\circ \phi_{t_{n-1}}^{\ell },F_{Y}\right]$ and the deformation field $\phi_{t_{n-1}}^{\ell }$ as input.

By stacking ConvLSTM blocks across *N* cascades, the SR-Module captures local deformation increments at each time step *t*_*n*_ and iteratively updates the global deformation field. The output increment $u_{t_{n}}^{\ell }$ is scaled by a factor $\frac{\Delta t}{h(\phi_{t_{n-1}}^{\ell })}$ and added to the previous deformation field $\phi_{t_{n-1}}^{\ell }$ to obtain $\phi_{t_{n}}^{\ell }$. This temporal integration mechanism enables smooth and progressive refinement of deformations across scales. To ensure symmetry, the SR-Module processes both the forward path (from moving to fixed image) and the backward path (from fixed to moving image) using shared-weight ConvLSTM blocks. In the backward path, the feature order is reversed to $\left[F_{Y},F_{X}\circ (\phi_{1}^{\ell }{)}^{-1}\right]$, ensuring consistent symmetric registration. Overall, the SR-Module reduces parameter redundancy, mitigates overfitting risk, and improves training efficiency, while maintaining flexibility to integrate future temporal modeling advancements.

### The MFDC Module

To guarantee strictly diffeomorphic registration, this study proposes the Multiscale Folding-aware Deformation Correction (MFDC) module. This module consists of two synergistic components: a coarse-to-fine folding-aware fusion and a localized geometry-based correction.

### Multiscale folding-aware correction

To mitigate severe folding regions and propagate smoothness from coarse to fine levels, this study adopts a multiscale fusion strategy based on folding-aware confidence weights. Let $\phi^{\left(r\right)}$ denote the deformation field at resolution level $r\in \{1,\frac{1}{2},\frac{1}{4},\frac{1}{8},\frac{1}{16}\}$, where *r* = 1 denotes the full-resolution field. At each location x∈Ω, this study computes the Jacobian determinant $J^{\left(1\right)}(x)=\det(\nabla \phi^{\left(1\right)}(x))$ and defines the folding region $ℱ=\{x|J^{\left(1\right)}(x)<0\}$. A Gaussian-smoothed folding-density map ρ(x) is then used to modulate the fusion weights, such that the full-resolution weight $W_{1}(x)$ is suppressed in folding-prone regions and redistributed to coarser levels via:


$$W_{1}(x)=(1-\mathrm{sigmoid}(k(\rho (x)-\tau )))\;W_{\text{folding}}(x)+\mathrm{sigmoid}(k(\rho (x)-\tau ))\;\varepsilon ,$$

$$W_{r}(x)=\beta_{r}(1-W_{1}(x)),\;r\in \left\{\frac{1}{2},\frac{1}{4},\frac{1}{8},\frac{1}{16}\right\}.$$

All weights are normalized such that $W_{1}(x)+\sum_{r}W_{r}(x)=1$, where the summation is taken over $r\in \{\frac{1}{2},\frac{1}{4},\frac{1}{8},\frac{1}{16}\}$. The fused deformation field is then defined as:


$$\phi_{\text{fused}}(x)=W_{1}(x)\;\phi^{\left(1\right)}(x)+\sum_{r}W_{r}(x)\;\phi^{\left(r\right)}(x).$$

where $W_{\text{folding}}(x)$ denotes a fold-aware confidence weight, τ is a folding-density threshold, *k* controls the slope of the sigmoid function, ε is a small constant, and $\beta_{r}$ are the coarse-level mixing coefficients. All coarse-resolution deformation fields $\phi^{\left(r\right)}$ are upsampled to the full resolution before fusion. Here, ρ(x) denotes the Gaussian-smoothed folding-density map, and the coefficients $\beta_{r}$ satisfy the normalization condition across coarse scales.

This fusion scheme allows coarse flows to dominate in regions with severe folding or ambiguity while preserving high-resolution details in smooth and reliable areas, thereby providing a strong initialization for subsequent local correction. Notably, when MFDC is integrated into single-scale registration backbones, the required multiscale hierarchy is constructed by applying successive downsampling and anti-aliasing filters to the predicted full-resolution deformation field, thereby extending the benefits of multiscale folding-aware fusion to any standard architecture.

### Geometry-based folding-correction

To further eliminate residual folding artifacts after fusion, this study adopts a polygon kernel-based correction mechanism, inspired in part by the geometric principles of star-shape priors as introduced in variational segmentation models [43]. This study first establishes the following Lemma:

**Lemma 1.** *Let P be a simple polygon (e.g., an octagon) whose kernel* ker(P) *is defined as the non-empty intersection of all half-planes formed by its edges. If* ker(P) *is non-empty, then there exists at least one interior point that forms consistently oriented triangles with every pair of consecutive vertices of P—i.e., P admits at least one configuration without folding.*

*Proof.* The kernel ker(P) is defined as the set of points from which every point on the boundary of *P* is visible; that is, for any p∈ker(P), the line segment connecting *p* to any point on the boundary of *P* lies entirely within *P* [44]. As the intersection of a finite number of half-planes defined by the polygon’s edges, ker(P) is necessarily convex [45].

Consider any p∈ker(P) and any consecutive vertex pair (*v*_*i*_, *v*_*i*+1_). The signed area of the triangle $△(p,v_{i},v_{i+1})$ must maintain a consistent sign for all such pairs. If any triangle were oriented oppositely, the visibility from *p* to the edge (*v*_*i*_, *v*_*i*+1_) would be obstructed, contradicting the definition of the kernel. Thus, any point p∈ker(P) serves as a folding-free interior reference, ensuring consistent local topology. □

**Fig 2:**
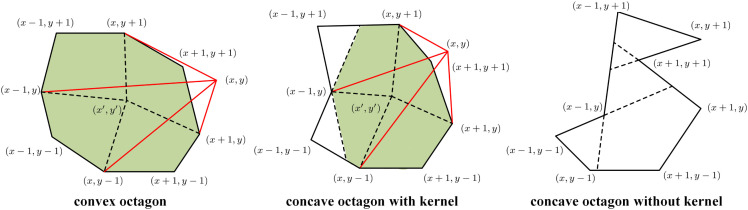
Illustration of local polygon types centered at a folding. Left: convex polygon; middle: concave polygon with a non-empty kernel; right: concave polygon without a kernel (empty kernel). The shaded regions represent the visible kernel, and red lines denote the visibility path from each vertex to the kernel area.

Fig 2 illustrates three representative neighborhood configurations: a convex polygon, a concave polygon with a kernel, and a concave polygon without a valid kernel, where local correction is infeasible.

Fig 2 provides a 2D schematic illustration of three representative local configurations: a convex polygon, a concave polygon with a non-empty kernel, and a concave polygon without a valid kernel, in which local correction is infeasible. This 2D example is used only for visualization and intuition. In the actual algorithm, the same kernel-validity principle is extended to the local 3D neighborhood around each folding voxel.

Based on the above lemma, and guided by the 2D illustration in Fig 2, the actual correction is performed in 3D. For each folding voxel x satisfying $\det(\nabla \phi_{\text{fused}}(x))<0$, this study considers its 26-connected neighborhood $N_{3}(x)$ and constructs a local geometric configuration from the deformed neighbors $\left\{\phi_{\text{fused}}(y)|y\in N_{3}(x)\right\}$. This study then evaluates whether the corresponding local configuration admits a valid kernel. If the kernel is non-empty, the folding is corrected by projecting $\phi_{\text{fused}}(x)$ to the centroid of that kernel. Otherwise, this study iteratively adjusts the most concave neighbor $v_{i}\in N_{3}(x)$, identified by angular deviation or coordinate variance, to promote a locally feasible configuration. If no update yields a recoverable kernel, the procedure returns to the Multiscale Folding-Aware Correction step. Through this iterative MFDC loop, residual folding can be progressively reduced and ultimately eliminated from the deformation field.

Algorithm 2 summarizes the implementation, which details the kernel verification steps, iterative concavity adjustments, and convergence criteria.

**Algorithm 2 MFDC correction procedure**

**S-1 Input:** The initial full-resolution deformation field $\phi^{\left(1\right)}$, multiscale deformation fields ${\left\{\phi^{\left(r\right)}\right\}}_{r\in \{1,\frac{1}{2},\frac{1}{4},\frac{1}{8},\frac{1}{16}\}}$, and the maximum number of correction iterations $T_{max}$.

**S-2 Multiscale Folding-Aware Fusion:** Compute the Jacobian determinant $J(x)\;=\;det(\nabla \phi^{\left(1\right)}(x))$. Generate confidence weights $W_{r}(x)$ based on the folding density ρ(x) and fuse.

$\begin{array}{c} \phi_{fused}(x)\;=\;\sum_{r}W_{r}(x)\;\cdot \;\phi^{\left(r\right)}(x) \end{array}$.

**S-3 Geometry-Based Correction Loop:** Iteratively refine $\phi_{fused}$ for *s* = 1 to $T_{max}$:

**S-3.1** Identify the set of folding points $F_{s}=\{x|det(\nabla \phi_{fused}(x))<0\}$.

**S-3.2** For each $x\;\in \;F_{s}$, construct a local configuration $P_{x}$ from the neighborhood $N_{3}(x)$.

• If $(P_{x})\;\neq \emptyset$, update $\phi_{fused}(x)$ to the centroid of $\left(P_{x}\right)$.

• If $(P_{x})\;=\;\emptyset$, identify the most concave neighbor $v^{*}\;\in \;N_{3}(x)$ via coordinate variance and shift $v^{*}$ toward its local kernel.

**S-3.3** Terminate if $F_{s}\;=\;\emptyset$ or if the number of residual foldings does not decrease.

**S-4 Output:** The final diffeomorphism-preserving field $\phi_{fused}$.

### Loss Function

The proposed loss system for DTC-Reg incorporates five elements: similarity loss, smoothness loss, Jacobian loss, cycle consistency loss, and control incremental constraint. The primary aim of similarity loss is to enhance the correlation between images. Conversely, the smoothness loss, Jacobian loss, cycle consistency loss, and control incremental constraint work together to maintain the smoothness and diffeomorphism of the registration grid.

### Similarity loss

This study employs the Normalized Cross-Correlation (NCC) [46] to measure similarity. The proposed method takes into account both the forward and backward registration steps, as well as the registration outputs at multiple scales (1≤ℓ≤L). The similarity loss is formally expressed as:


$$\begin{array}{cc} {ℒ}_{sim}= & -\sum_{\ell =1}^{L}\lambda_{1}^{\ell }(\text{NCC}(X\circ 𝒫(\phi_{1}^{\ell }),Y)\\ & \;+\text{NCC}(X,Y\circ 𝒫((\phi_{1}^{\ell }{)}^{-1}))), \end{array}$$

in which *X* and *Y* represent the moving and fixed images, respectively. *L* stands for the total number of scales, $\phi_{1}^{\ell }$ and $(\phi_{1}^{\ell }{)}^{-1}$ correspond to the deformations generated by DTC-Reg in both forward and reverse directions across multiple scales. $\lambda_{1}^{\ell }$ is a parameter that is used to weigh the importance of similarity loss on different scales. The term 𝒫 refers to the trilinear upsampling operator, where the deformation fields on scales ℓ=5,4,3,2 are upsampled to the full scale for loss computation.

### Jacobian loss

In the DTC-Reg framework, this study incorporates a Jacobian loss ${ℒ}_{Jdet}$ to ensure the maintenance of the diffeomorphism characteristic of the deformation field. This loss is applied to both the forward deformation field $\phi_{1}^{\ell }$ and its inverse $(\phi_{1}^{\ell }{)}^{-1}$ at each level. It is determined by evaluating the negative values of the Jacobian determinant for each point on the registration grid, employing the Rectified Linear Unit (ReLU) activation function:


$${ℒ}_{Jdet}=\sum_{\ell =1}^{L}\sum_{x\in \Omega }\left(\text{ReLU}(-J_{\phi_{1}^{\ell }}(x))+\text{ReLU}(-J_{(\phi_{1}^{\ell }{)}^{-1}}(x))\right).$$

where $J_{\phi_{1}^{\ell }}(x)=\det(\nabla \phi_{1}^{\ell }(x))$ and $J_{(\phi_{1}^{\ell }{)}^{-1}}(x)=\det(\nabla (\phi_{1}^{\ell }{)}^{-1}(x))$ denote the Jacobian determinants of the forward and inverse deformation fields, respectively.

### Smooth loss

To ensure that the deformation fields remain smooth across all levels, DTC-Reg incorporates a $\ell_{2}$ regularization term into the gradient fields of the deformations. The smoothness loss can be defined as:


$${ℒ}_{reg}=\sum_{\ell =1}^{L}\sum_{x\in \Omega }\left(∥\nabla \phi_{1}^{\ell }\left(x\right){∥}_{2}^{2}+∥\nabla (\phi_{1}^{\ell }{)}^{-1}\left(x\right){∥}_{2}^{2}\right),$$

which ensures the smoothness of the forward and backward deformation fields.

### Cycle consistency loss

Since DTC-Reg performs a symmetric registration path and generates a pair of two-way deformation fields simultaneously, it enables the creation of a cycle consistency loss based on these fields. The loss of cycle consistency can be expressed as:


$${ℒ}_{cycle}=-\text{NCC}(X,X\circ (\phi_{1}\circ \phi_{1}^{-1}))-\text{NCC}(Y,Y\circ (\phi_{1}^{-1}\circ \phi_{1})).$$

This study emphasizes that the symmetric architecture and the cycle consistency loss serve distinct yet complementary purposes. While the symmetric design provides a structural prior by coupling the forward and inverse paths to facilitate joint learning, the cycle consistency loss acts as an explicit output constraint to prevent accumulated drift and enforce mutual consistency through composition.

### Control incremental constraint

According to the algorithm proposed in [12], the control increment field u(ϕ(x,t)) in the DTC-Reg framework should satisfy equation (4). To ensure consistency with the diffeomorphic theory, an additional constraint is added to the total loss function, which is expressed as:


$$\begin{array}{cc} {ℒ}_{cic}= & \sum_{\ell =1}^{L}\{\left|\text{div}(u(\phi^{\ell }))+\frac{\partial h(\phi^{\ell },t)}{\partial t}\right|\\ & +\left|\text{div}(u((\phi^{\ell }{)}^{-1}))+\frac{\partial h((\phi^{\ell }{)}^{-1},t)}{\partial t}\right|\}. \end{array}$$

where ℓ indexes the scale level, *t* denotes the temporal variable, $\phi^{\ell }$ represents the deformation field at scale ℓ, and div(·) denotes the spatial divergence operator.

### Total Loss

The total training loss of the proposed DTC-Reg can be expressed as:


$$ℒ(X,Y)={ℒ}_{sim}+\lambda_{2}{ℒ}_{Jdet}+\lambda_{3}{ℒ}_{reg}+\lambda_{4}{ℒ}_{cycle}+\lambda_{5}{ℒ}_{cic},$$

where $\lambda_{1}^{\ell }$, $\lambda_{2}$, $\lambda_{3}$, $\lambda_{4}$ and $\lambda_{5}$ are the weights of the similarity loss on each scale ℓ, Jacobian loss, smoothness loss, cycle consistency loss, and control incremental constraint loss, respectively.

## Experiment

### Experimental settings

#### Inter-patient Brain MRI Registration.

Experiments for aligning brain MRI scans of different patients were conducted using the OASIS-v1 dataset [47]. For fair comparison and reproducibility, this study utilized the standard pre-processed version of the dataset provided by Hoopes et al. [24]. The dataset comprises 414 T1-weighted MRI images and their segmentation labels. The preprocessing pipeline, as detailed in [24], utilized FreeSurfer [48] for motion correction, skull stripping, affine spatial normalization, and subcortical segmentation. No additional manual intervention was applied to the provided data in the experiments. The images were resized from dimensions 160 × 192 × 224 to 160 × 160 × 192. The dataset division included 255 images for the training set, nine for validation, and 150 for testing. During training, image pairs for alignment were randomly selected, resulting in 64,770 pairs. For validation, one image was fixed and the remaining eight were treated as moving images, forming eight pairs of validation images. During testing, five images from the test set were chosen, with one randomly set as fixed image per iteration. Moving images were selected from the remaining 145, generating 725 test image pairs. The segmentation labels covered 35 anatomical structures to assess the accuracy of the registration.

### Patient-to-atlas Brain MRI registration

The dataset used in this study, provided by Chen et al. [37], was used to align brain MRI scans between patient data and an atlas. It includes 576 T1-weighted MRI brain scans in addition to an atlas image. The moving images originated from the IXI dataset, while the fixed images were obtained from the research by Kim et al. [27]. This dataset was divided into training, validation, and testing subsets with ratios of 403:58:115 (7:1:2). Each image was resized to dimensions of 160 × 160 × 192. To assess registration accuracy, segmentation was performed in 30 different anatomical regions.

### Few-Shot Dataset MRI Registration

For the Few-Shot MRI Registration task, the Mindboggle101 dataset [49] was utilized, focusing on the NKI-RS-22, NKI-TRT-20, and OASIS-TRT-20 subsets, which together provided 62 T1-weighted brain MRI scans. Originally aligned in the MNI152 space with a resolution of 182 × 218 × 182, these images were subsequently resized to dimensions of 160 × 192 × 160. The dataset was divided into a training set of 50 images and a testing set of 12 images. During training, this study randomly selected pairs from the training set to generate 2,450 pairs of training images. In the testing phase, one image served as the fixed reference, while the other 11 were used as moving images, forming 11 test image pairs.

### Comparison methodology

The proposed DTC-Reg model is evaluated against a traditional variational method and several representative deep learning approaches. The selected baselines include SyN [11], VoxelMorph [24], VoxelMorph-Diff [25], TransMorph [37], TransMorph-Diff [37], SYM-net [28], VTN [26], LapIRN [50], and RDP [51]. For all comparison methods, the optimal parameter configurations reported in their original publications were adopted.

### Evaluation metrics

The performance of the registration was assessed by examining the anatomical features of the aligned images using the Dice similarity coefficient (DSC) and the Hausdorff distance (HD). A quantitative evaluation involved comparing the mean and standard deviation of DSC and HD for the designated anatomical features among all patients. The structural similarity index (SSIM) was employed to evaluate the similarity between the fixed image and the registered image. Furthermore, the number of voxels with non-positive Jacobian determinants in the deformation field, denoted as MFN (Mean Folding Number), was used to measure the folding ratios of the registration field.

### Implementation

The Python language (version 3.11.5) and the PyTorch deep learning framework (version 2.1.2) were utilized on a Linux OS (Ubuntu 22.04.1 LTS). The hardware setup included a 12th-gen Intel (R) Core (TM) i7-12700F CPU and a single NVIDIA GeForce RTX 4090 GPU. The loss weights were selected by empirical tuning on the validation split. The loss weights were initially set using commonly adopted magnitudes in prior learning-based registration studies and were subsequently adjusted to balance registration accuracy, regularization, and topology-related constraints. For consistency, the same set of weights was used across all datasets and experiments. For DTC-Reg, the learning rate was set to 10^−4^ and a batch size of 1 was used. The weights of the loss function were fixed as $\lambda_{1}=0.8$, $\lambda_{2}=1\times 10^{-4}$, $\lambda_{3}=1$, $\lambda_{4}=0.1$, and $\lambda_{5}=0.1$.

### Comparison with baseline methods

To evaluate the effectiveness of the proposed DTC-Reg framework and the specific contribution of the MFDC module, this study conducted a comprehensive comparison against representative registration approaches. These baselines include classical methods (SyN), deformation-prediction CNNs (VoxelMorph), diffeomorphic variants (VoxelMorph-Diff), Transformer-based frameworks (TransMorph), and their diffeomorphic counterparts (TransMorph-Diff), as well as symmetric networks (SYM-net) and recent multiscale/pyramidal models (VTN, LapIRN, and RDP). For fairness, all learning-based baselines were retrained from scratch using the same dataset splits and preprocessing pipeline as DTC-Reg. This study used the official implementations and followed the recommended hyperparameter settings in the original publications, while keeping the training and evaluation protocol consistent across all methods.

Table 1 summarizes the quantitative performance of the proposed full DTC-Reg framework against these baselines on the OASIS-v1, IXI, and Mindboggle101 datasets. As a coarse-to-fine multiscale framework, DTC-Reg demonstrates superior registration capability, achieving competitive or state-of-the-art accuracy across all metrics. Crucially, unlike conventional CNNs (e.g., VoxelMorph) or Transformer-based methods which suffer from severe topological folding (high MFN), DTC-Reg consistently maintains strictly diffeomorphic transformations (MFN = 0). Furthermore, compared to existing diffeomorphic variants that often sacrifice alignment accuracy to satisfy topological constraints, the proposed method successfully resolves the accuracy-topology trade-off. It also outperforms other modern multiscale baselines (e.g., LapIRN and RDP) in terms of DSC and Hausdorff distance, verifying the efficacy of the proposed architectural design.

**Table 1:** Quantitative comparison of registration baselines and their MFDC-enhanced versions. Results are reported as mean (standard deviation). For each dataset, higher DSC and SSIM and lower HD and MFN indicate better performance. Indented rows represent models after integrating the proposed MFDC module. Arrows (↑, ↓) denote significant changes relative to the baseline.

Model	OASIS-v1				IXI				Mindboggle101			
	DSC (%)	HD	SSIM	MFN	DSC (%)	HD	SSIM	MFN	DSC (%)	HD	SSIM	MFN
Initial	60.06 (6.11)	3.58 (0.85)	0.68 (0.02)	–	40.64 (3.50)	6.48 (0.67)	0.62 (0.01)	–	39.25 (1.99)	6.68 (0.51)	0.65 (0.01)	–
SyN	75.90 (3.05)	2.21 (0.48)	0.82 (0.02)	0	65.93 (3.83)	4.50 (0.78)	0.80 (0.02)	0.3	55.00 (0.97)	5.60 (0.34)	0.81 (0.01)	0
VM	76.80 (3.43)	2.39 (0.62)	0.91 (0.01)	66508	72.91 (2.62)	3.69 (0.67)	0.88 (0.01)	95650	59.99 (1.46)	5.76 (0.42)	0.93 (0.01)	83588
VM + MFDC	74.52 (3.55)↓	2.72 (0.65)↑	0.88 (0.02)↓	0↓	71.05 (2.80)↓	3.98 (0.75)↑	0.85 (0.02)↓	0↓	58.12 (1.60)↓	6.05 (0.48)↑	0.91 (0.01)↓	0↓
VM-Diff	72.83 (4.65)	2.56 (0.65)	0.80 (0.02)	2.8	70.51 (2.73)	3.27 (0.50)	0.75 (0.02)	0	53.44 (1.32)	5.83 (0.41)	0.81 (0.01)	12.0
VM-Diff + MFDC	72.83 (4.65)	2.56 (0.65)	0.80 (0.02)	0↓	70.51 (2.73)	3.27 (0.50)	0.75 (0.02)	0	53.42 (1.35)↓	5.85 (0.41)↑	0.81 (0.01)	0↓
TM	78.41 (4.81)	2.04 (0.49)	**0.93 (0.01)**	30484	74.56 (2.12)	3.03 (0.42)	**0.89 (0.02)**	73797	57.85 (2.72)	5.94 (0.41)	**0.94 (0.01)**	94097
TM + MFDC	77.25 (4.50)↓	2.25 (0.55)↑	0.90 (0.01)↓	0↓	73.10 (2.30)↓	3.28 (0.50)↑	0.86 (0.02)↓	0↓	56.45 (2.60)↓	6.22 (0.45)↑	0.92 (0.01)↓	0↓
TM-Diff	74.90 (3.79)	2.26 (0.46)	0.89 (0.01)	0	72.09 (3.11)	3.21 (0.51)	0.75 (0.02)	0	52.11 (1.11)	5.71 (0.36)	0.78 (0.01)	10.3
TM-Diff + MFDC	74.90 (3.79)	2.26 (0.46)	0.89 (0.01)	0	72.09 (3.11)	3.21 (0.51)	0.75 (0.02)	0	52.10 (1.11)↓	5.72 (0.36)↑	0.78 (0.01)	0↓
SYM-net	79.09 (2.67)	2.10 (0.50)	0.92 (0.01)	50	74.93 (2.03)	3.02 (0.48)	0.88 (0.01)	24	57.45 (1.45)	5.94 (0.44)	0.90 (0.01)	46
SYM-net + MFDC	79.07 (2.68)↓	2.13 (0.51)↑	0.92 (0.01)	0↓	74.90 (2.05)↓	3.05 (0.49)↑	0.87 (0.01)↓	0↓	57.43 (1.48)↓	5.95 (0.45)↑	0.89 (0.01)↓	0↓
VTN	77.45 (3.10)	2.18 (0.45)	0.91 (0.01)	4520	73.80 (2.40)	3.15 (0.42)	0.88 (0.01)	5210	58.50 (1.65)	5.68 (0.38)	0.92 (0.01)	6140
VTN + MFDC	76.95 (3.20)↓	2.25 (0.48)↑	0.89 (0.01)↓	0↓	73.42 (2.50)↓	3.22 (0.43)↑	0.86 (0.01)↓	0↓	57.90 (1.70)↓	5.80 (0.42)↑	0.90 (0.01)↓	0↓
LapIRN	78.60 (2.75)	2.08 (0.42)	0.92 (0.01)	120	75.10 (2.15)	2.92 (0.39)	0.88 (0.01)	150	59.50 (1.50)	5.55 (0.36)	0.93 (0.01)	180
LapIRN + MFDC	78.52 (2.78)↓	2.10 (0.45)↑	0.91 (0.01)↓	0↓	75.02 (2.20)↓	2.94 (0.41)↑	0.87 (0.01)↓	0↓	59.42 (1.55)↓	5.57 (0.38)↑	0.92 (0.01)↓	0↓
RDP	78.20 (2.60)	2.12 (0.43)	0.92 (0.01)	210	74.60 (2.20)	3.01 (0.40)	0.88 (0.01)	240	59.10 (1.55)	5.62 (0.37)	0.92 (0.01)	290
RDP + MFDC	78.12 (2.60)↓	2.15 (0.44)↑	0.91 (0.01)↓	0↓	74.52 (2.25)↓	3.04 (0.42)↑	0.87 (0.01)↓	0↓	59.02 (1.60)↓	5.64 (0.39)↑	0.91 (0.01)↓	0↓
**DTC-Reg (proposed)**	**79.88 (2.50)**	**2.00 (0.41)**	**0.91 (0.01)**	**0**	**76.60 (1.79)**	**2.77 (0.45)**	**0.85 (0.02)**	**0**	**61.22 (2.11)**	**5.42 (0.64)**	**0.91 (0.01)**	**0**

**Fig 3:**
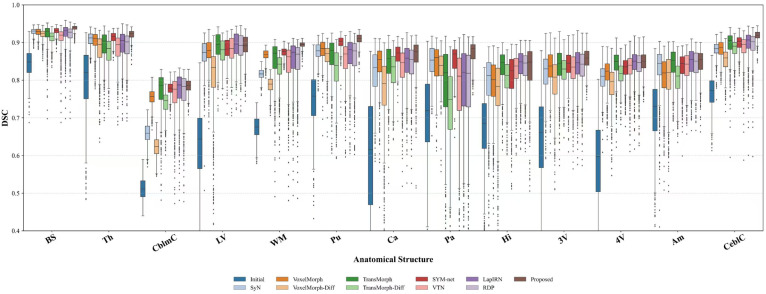
Regional registration accuracy comparison on the OASIS-v1 dataset. Comparison of DSC scores for each anatomical region between state-of-the-art methodologies and the proposed approach. Left and right brain hemispheres are combined into a single region for clarity. Analyzed structures include: brain stem (BS), thalamus (Th), cerebellar cortex (CblmC), lateral ventricle (LV), cerebellar white matter (WM), putamen (Pu), caudate (Ca), pallidum (Pa), hippocampus (Hi), 3rd ventricle (3V), 4th ventricle (4V), amygdala (Am), CSF (CSF), and cerebral cortex (CeblC).

As illustrated in Fig 3, the proposed method outperforms the baseline methods across the majority of anatomical structures. The quantitative results indicate that the proposed framework consistently achieves higher median DSC scores and more stable distributions with fewer outliers. These observations validate the robustness of the proposed method and demonstrate its superior registration accuracy compared to existing approaches.

**Fig 4:**
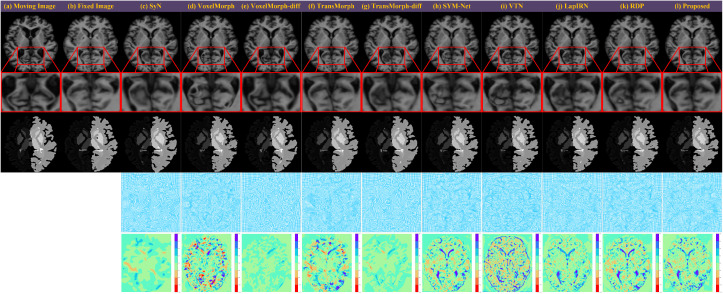
Qualitative registration results on a representative OASIS-v1 sample. Rows from top to bottom represent: warped images, zoomed regions of interest (ROIs), warped segmentation labels, deformation grids, and Jacobian determinant maps. Standard learning-based methods, such as VoxelMorph (d) and TransMorph (f), achieve sharp intensity matching but exhibit topological folding, visible as noise in Jacobian determinant maps. Diffeomorphic variants (e, g) enforce topology but over-smooth anatomical details. Recent multiscale baselines VTN (i), LapIRN (j), and RDP (k) improve regularity but miss fine-grained structural alignments. In contrast, DTC-Reg (l) preserves high-fidelity anatomical details while ensuring a strictly regular, folding-free deformation field.

Fig 4 presents a qualitative comparison on a representative OASIS-v1 sample, showing warped images, zoomed regions of interest (ROIs), deformation grids, and Jacobian determinant maps. Standard learning-based methods, such as VoxelMorph (d) and TransMorph (f), achieve sharp intensity matching but suffer from severe topological violations, manifested as highly irregular grids and noisy Jacobian determinant maps (indicating folding). While their diffeomorphic variants (e, g) successfully enforce topology, they tend to over-smooth the deformation, leading to blurred anatomical details in the zoomed ROIs. Among the recent multiscale baselines, VTN (i) still exhibits minor topological irregularities (visible as scattered noise in the Jacobian determinant map), whereas LapIRN (j) and RDP (k) produce smoother fields but occasionally miss fine-grained structural alignments compared to the proposed method. In contrast, DTC-Reg (l) achieves the optimal balance: it preserves the sharp anatomical fidelity characteristic of high-capacity models while ensuring a strictly regular, folding-free deformation field, visually outperforming both standard and multiscale baselines.

### MFDC as a plug-and-play correction module

To rigorously isolate the specific contribution of the MFDC module and verify its versatility, this study integrated it as a plug-and-play post-processing unit into a diverse range of registration backbones, spanning standard CNNs, Transformers, and multiscale pyramidal models. As summarized in Table 1, the quantitative results demonstrate that MFDC functions as a robust, backbone-agnostic correction mechanism. It systematically eliminates topological violations—reducing the MFN to zero across all experiments—regardless of the predictor’s initial folding severity, while maintaining or even improving the DSC score by stabilizing the deformation fields. Regarding computational efficiency, at inference, MFDC is triggered only in local neighborhoods around detected folding voxels (negative-Jacobian regions) rather than over the full field; in the experiments the number of such voxels is small (typically ~4–450 per volume), and thus the added runtime overhead is minimal compared with the backbone.

### Comprehensive ablation analysis of the DTC-Reg framework

A systematic ablation study was conducted on the OASIS-v1 dataset to evaluate the individual contributions of recurrent units, variational loss formulations, and the internal mechanisms of the MFDC module. As summarized in Table 2, the choice of temporal recurrent units is foundational to the framework’s performance. Replacing the ConvLSTM with ConvGRU (a) or a standard ResNet architecture (b) results in a substantial decline in both alignment accuracy and topological regularity. This confirms that the long-term dependency modeling of ConvLSTM is superior for capturing the evolution of deformation fields within the temporal refinement cascade. The individual contributions of the variational loss terms are equally critical. Specifically, the removal of Cycle Loss (d), Smoothness Loss (e), or Jacobian Loss (f) leads to a drastic surge in folding artifacts, with the MFN soaring to 94.21, 289.18, and 379.98, respectively. Furthermore, the absence of the Control Incremental Constraint (${ℒ}_{cic}$) (i) causes the MFN to exceed 122, underscoring the necessity of the homotopy-based variational formulation for guiding diffeomorphic flows. Architectural features such as the symmetric path (g) and pre-warped (deformed) features (h) also prove essential, as their removal diminishes DSC and compromises grid stability. To further scrutinize the proposed MFDC module, its internal components were evaluated: multiscale fusion and geometric correction. While the baseline S-DTC-Reg (j) achieves a high DSC of 79.89%, it retains residual folding (*MFN* = 2.80). Integrating multiscale fusion only (l) or geometric correction only (m) reduces the folding count to 1.12 and 0.35, respectively, but fails to ensure absolute topological integrity. Crucially, only the Full MFDC (k)—which synergistically combines hierarchical weight redistribution with localized polygon-kernel projection—achieves absolute topological preservation (*MFN* = 0) while maintaining optimal alignment fidelity. This demonstrates that DTC-Reg effectively reconciles the accuracy-topology trade-off, delivering a strictly diffeomorphic transformation through multi-level geometric constraints.

**Table 2:** Quantitative assessments of ablation studies on the OASIS-v1 dataset. Comparison of architectural components and the internal mechanisms of the MFDC module. Values are reported as mean ± standard deviation. Bold indicates the final proposed framework with strict diffeomorphism.

Variants	DSC (%) ↑	MFN ↓
**S-DTC-Reg (Architecture & Losses)**		
(a) r/ Conv-GRU	70.42 ± 6.69	56.69 ± 12.74
(b) r/ ResNet	78.16 ± 2.75	1.50 ± 4.21
(c) r/ TransMorph	78.72 ± 2.15	3.59 ± 2.35
(d) w/o Cycle Loss	79.29 ± 3.00	94.21 ± 33.55
(e) w/o Smooth Loss	79.52 ± 3.39	289.18 ± 54.89
(f) w/o Jacobian Loss	79.88 ± 3.32	379.98 ± 78.45
(g) w/o symmetric path	79.05 ± 3.23	96.87 ± 34.94
(h) w/o deformed features	78.75 ± 2.45	2.78 ± 4.63
(i) w/o ${ℒ}_{cic}$	79.89 ± 2.45	122.78 ± 15.45
(j) Baseline S-DTC-Reg (no MFDC)	79.89 ± 2.48	2.80 ± 1.36
**DTC-Reg (Internal MFDC Mechanisms)**		
(k) Full MFDC (multiscale + geometric)	**79.88 ± 2.50**	**0 ± 0**
(l) Multiscale fusion only	79.88 ± 2.50	1.12 ± 1.90
(m) Geometric correction only	79.88 ± 2.50	0.35 ± 0.92

### Effect of Cascade Depth

The SR-Module in DTC-Reg refines the deformation field through a hierarchy of temporal cascades, where each level integrates incremental velocity updates under the homotopy constraint. To better understand how cascade depth influences registration quality, the number of temporal stages *N* was varied while keeping the number of spatial scales fixed.

Table 3 reports the results for configurations ranging from 5 × 1 to 5 × 6. Increasing the number of cascades brings two benefits. First, deeper cascades allow more gradual velocity integration, producing smoother and more accurate deformations. This is reflected by progressive improvements in DSC and reductions in HD as *N* increases from 1 to 4. Second, deeper cascades reduce folding accumulation by distributing refinement steps across multiple temporal updates rather than enforcing large deformations at once.

**Table 3:** Analysis of cascade depth in the SR-Module on the OASIS-v1 dataset. Values are reported as mean (standard deviation). Higher DSC and SSIM, and lower HD and MFN indicate better performance. Results are shown for different numbers of temporal cascades *N.*

Cascade ℓ×N	DSC (%) ↑	HD ↓	SSIM ↑	MFN ↓	Params
5 × 1	76.21 (3.62)	2.31 (0.53)	0.837 (0.02)	0 (0)	0.77M
5 × 2	78.24 (2.98)	2.12 (0.47)	0.872 (0.01)	0 (0)	0.87M
5 × 3	78.92 (2.94)	2.08 (0.46)	0.887 (0.01)	0 (0)	0.97M
5 × 4	79.88 (2.50)	**2.00 (0.41)**	0.910 (0.01)	0 (0)	1.07M
5 × 5	79.89 (2.87)	2.05 (0.45)	0.904 (0.01)	0 (0)	1.18M
5 × 6	**79.92 (2.87)**	2.03 (0.46)	**0.917 (0.01)**	0 (0)	1.28M

However, when the cascade becomes deeper than four stages (N≥5), the performance gains become marginal relative to the increased model complexity. This aligns with theoretical expectations: finer temporal granularity helps, but excessive stacking introduces optimization noise and additional interpolation, which can destabilize fine-scale updates. In practice, *N* = 4 provides the best trade-off between accuracy, stability, and parameter efficiency, and is therefore adopted as the default setting in all experiments.

## Internal network visualization

**Fig 5:**
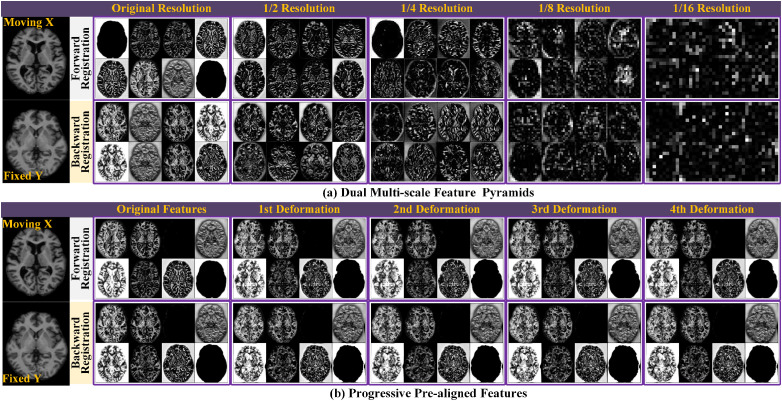
Visualization of internal feature maps and progressive deformation. **(a) Dual multiscale feature pyramids:** Eight representative 2D slice feature maps randomly selected from five scales within the two feature pyramids. **(b) Progressive feature registration:** Evolution of feature maps during registration at the finest scale, demonstrating how initial features in DTC-Reg are gradually deformed to achieve alignment.

Fig 5(a) presents an illustration of the progressive feature-deforming volumes within dual multiscale feature pyramids, with eight 2D slices extracted at each scale. The features obtained closely match the theoretical forecasts. To enhance interpretability, two separate networks are utilized to extract features from the two images, rather than using a single U-Net to extract features and learn the deformation field. At the coarse scale, the extracted features represent the global structure of the original image, whereas at the fine scale, they capture finer local details. Fig 5(b) shows the step-by-step deformation of features throughout multi-moment registration at the full scale. Starting with the initial features, they are deformed four times through the cascaded ConvLSTM registration module, with each registration stage aligning the features appropriately.

**Fig 6:**
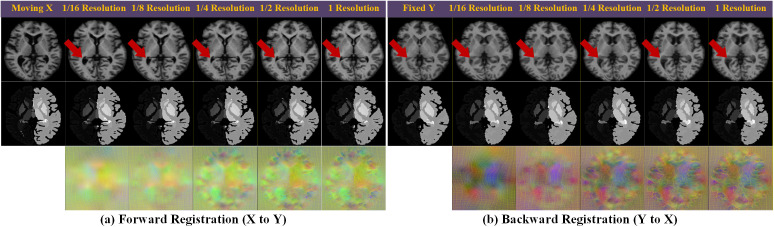
Visualizations of multiscale progressive registration within DTC-Reg. Rows from top to bottom represent: the original image, segmentation labels, and the registration grid. The sequence from left to right in panels (a) and (b) illustrates the original input followed by intermediate registered results at 1/16, 1/8, 1/4, 1/2, and full resolution, respectively. Each scale demonstrates the incremental refinement of both anatomical structures and the deformation grid.

The intermediate steps of multiscale registration are depicted in Fig 6. Beginning with a 1/16 resolution, the coarser scale helps approximate the deformation direction, while the finer scale refines the deformation of finer details. As the registration progresses, the source image aligns more closely with the fixed image, where the low-resolution grid shows only a general deformation, and higher-resolution grids acquire finer details with minimal folding.

**Fig 7:**
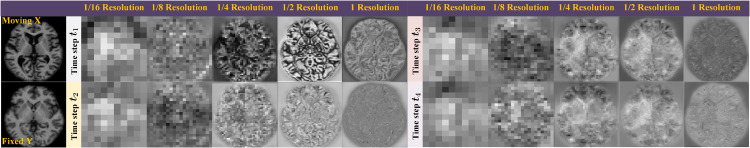
Visualizations of internal memory features across scales and time steps. The images display gray-level representations of the memory features *c*, obtained by averaging the values across its three channels. The visualization demonstrates the progressive evolution of memory states within the ConvLSTM structure across multiple SR-Module scales and sequential temporal increments, illustrating how registration information is accumulated and refined.

Fig 7 illustrates the visualization of the memory feature *c* in four distinct time steps on each scale within DTC-Reg. The memory feature is composed of three channels, and the results shown are the average of these channels. Fig 7 clearly demonstrates that the memory feature starts at the coarsest scale in the registration path, capturing the registration process incrementally and refining it to achieve the final prediction. The memory feature conveys details at various levels, suggesting that the ConvLSTM structure contributes to the registration path, aligning with theoretical expectations. This use of ConvLSTM-based temporal modeling for registration is not only feasible but also interpretable.

## Conclusion

This study presents DTC-Reg, a novel framework designed for symmetric diffeomorphic registration of medical images. The principal innovation lies in the integration of deep learning with the mathematical mechanisms of homotopy continuation, representing diffeomorphic registration as a continuous, time-evolving process across multiple spatial scales. To address the persistent challenge of topological violations in learning-based methods, this study introduced the Multiscale Folding-aware Deformation Correction (MFDC) module. Unlike conventional regularization techniques that often sacrifice significant alignment accuracy for regularity, the proposed MFDC module effectively decouples topological preservation from deformation prediction. Comprehensive experiments on three 3D brain MRI benchmarks demonstrate that DTC-Reg achieves state-of-the-art accuracy while maintaining strictly regular, folding-free deformation fields (*MFN* = 0). Notably, the MFDC module proves to be a robust, backbone-agnostic correction unit that eliminates folding with minimal loss in registration fidelity, resolving the long-standing accuracy-topology trade-off. The results demonstrate that combining temporal cascade architectures with geometric correction strategies offers a superior pathway for generating biologically plausible transformations in clinical applications. Although the experiments focus on brain MRI, DTC-Reg is not modality-specific and is potentially applicable to other modalities (e.g., CT) and organs (e.g., lung and liver). In these settings, performance may be affected by modality-dependent intensity distributions, lower soft-tissue contrast, organ motion (e.g., respiration), and increased anatomical variability. Importantly, the topology-preserving mechanism is expected to transfer directly because MFDC enforces geometric regularity on the estimated deformation field rather than relying on modality-specific appearance cues.
